# Epidemiological Determinants of Urolithiasis Recurrence: A Retrospective Cohort Study Integrating Stone Composition and Behavioral Factors

**DOI:** 10.3390/epidemiologia7040093

**Published:** 2026-07-03

**Authors:** Marius Ivănuță, Dragoș Puia, Mihaela Corlade-Andrei, Ana-Maria Ivănuță, Bogdan Doroftei, Octavia Petrila, Mihaela Nikolic, Cătălin Pricop

**Affiliations:** 1 Grigore T. Popa University of Medicine and Pharmacy Iasi, 700115 Iasi, Romania; marius.ivanuta@umfiasi.ro (M.I.); bogdan.doroftei@umfiasi.ro (B.D.); catalin.pricop@umfiasi.ro (C.P.); 2 Department of Urology, “Dr. C.I. Parhon” Clinical Hospital, 700503 Iasi, Romania; 3 Center for Morphological and Spectroscopic Analysis of Urinary Stones “Michel Daudon”, 700503 Iasi, Romania; 4 Emergency Care Department, “Sf. Spiridon” County University Emergency Hospital, 700111 Iasi, Romania; anamariaion92@yahoo.com; 5 Origyn Fertility Center, Palace Street No. 3C, 700032 Iasi, Romania; 6 Clinical Hospital of Obstetrics and Gynecology “Cuza Voda”, Cuza Voda Street No. 34, 700038 Iasi, Romania; 7 Department of Radiology, “Dr. C.I. Parhon” Clinical Hospital, 700503 Iasi, Romania; ociuhodaru@yahoo.com; 8 Department of Physiology, University of Life Sciences Ion Ionescu de la Brad, 700490 Iasi, Romania; mihaela_nikolic@yahoo.com

**Keywords:** urolithiasis, stone recurrence, stone composition, uric acid stones, calcium oxalate, risk factors, survival analysis, fluid intake

## Abstract

**Background**: Urolithiasis is a common condition characterized by a high risk of recurrence, although this risk varies substantially between patients. The present study aimed to quantify incidence, identify determinants, and model recurrence risk between stone composition and recurrence, and to assess the role of selected clinical and behavioral factors in a cohort of patients with confirmed stone clearance. **Methods**: This retrospective cohort study included adult patients with urolithiasis managed between 2017 and 2021 in a specialized referral center. Only patients with confirmed stone-free status and available follow-up were included. Stone composition was determined using morpho-spectroscopic analysis. Clinical and behavioral variables were collected from medical records. Recurrence was defined as a new stone event during follow-up. Time-to-event analysis was performed using Kaplan–Meier estimates and Cox proportional hazards models. **Results**: A total of 962 patients were included, with a median follow-up of over three years. During this period, recurrence occurred in approximately one-third of patients. Uric acid stones were associated with a higher risk of recurrence compared with calcium oxalate monohydrate stones, while the effect size was moderate. Previous stone history and low fluid intake were also independently associated with recurrence. Other dietary variables did not retain statistical significance after adjustment. **Conclusions**: Recurrence in urolithiasis appears to reflect the combined effect of compositional, clinical, and behavioral factors. While certain variables are associated with increased risk, no single determinant fully explains recurrence patterns. These findings support a more individualized approach to risk assessment and follow-up.

## 1. Introduction

Urolithiasis is a common condition with a growing global burden, affecting up to 10–15% of the population during their lifetime. Its incidence has increased steadily over recent decades, a trend attributed to changes in lifestyle, dietary habits, and the rising prevalence of metabolic disorders. Beyond the acute clinical manifestations, urinary stone disease is characterized by a high propensity for recurrence, with more than half of patients developing a new episode within 10 years after the initial event [1,2].

Recurrence of kidney stone disease is highly heterogeneous. While some patients experience only a single episode, others develop recurrent or even rapidly progressive disease, requiring repeated interventions and long-term monitoring. The risk of recurrence is influenced by a wide range of factors, including demographic characteristics, metabolic abnormalities, and prior stone history. Notably, the likelihood of recurrence increases with the number of previous stone episodes, reflecting the cumulative nature of lithogenic risk [3,4].

Stone formation is a complex and multifactorial process involving urinary supersaturation, crystal nucleation, growth, and aggregation, modulated by both promoting and inhibitory factors. In this context, stone composition represents an important surrogate of the underlying pathophysiological mechanisms. Moreover, previous studies have suggested that certain stone types, particularly uric acid and infection-related calculi, may be associated with a higher risk of recurrence [5,6]. In addition to compositional factors, a broad range of clinical and behavioral variables contribute to recurrence risk. Meta-analytic data have identified multiple predictors, including body mass index, metabolic comorbidities, prior stone history, and dietary patterns, although the strength and consistency of these associations vary across studies. Importantly, many of these factors are potentially modifiable, highlighting the relevance of individualized preventive strategies [7,8]. Despite these advances, the clinical interpretation of recurrence in urolithiasis remains challenging, largely due to heterogeneity in its definition and measurement. Recurrence has been variably defined, ranging from symptomatic stone events to radiographic findings such as new stone formation or growth of residual fragments, limiting comparability across studies and hindering meaningful interpretation of recurrence patterns. Moreover, relatively few studies have integrated stone composition with clinical and behavioral factors within a single, well-characterized cohort.

Previous studies have evaluated recurrence risk in urolithiasis using different definitions of recurrence and heterogeneous follow-up strategies. In addition, stone composition, clinical history, and behavioral factors have not always been assessed together within the same analytical framework. In this context, the present study aimed to provide additional data from a cohort of patients with documented stone clearance before follow-up, in whom stone composition was assessed by morpho-spectroscopic analysis and recurrence was evaluated using time-to-event methods.

The present study aimed to assess the association between stone composition and recurrence of urolithiasis in a cohort of patients managed in a specialized referral center. In addition, we sought to evaluate the contribution of selected clinical and behavioral factors to recurrence risk using time-to-event analysis.

## 2. Materials and Methods

### 2.1. Study Design and Setting

This retrospective cohort study was conducted at the “Dr. C. I. Parhon” Hospital, within the “Michel Daudon” Center for Morphological and Spectroscopic Analysis of Reno-Ureteral Lithiasis, a tertiary referral center for the management of urinary stone disease. Patients were included over a defined accrual period between January 2017 and December 2021 and were subsequently followed until April 2026. The study is reported in accordance with the STROBE guidelines.

### 2.2. Study Population

Adult patients diagnosed with urolithiasis during the study period were considered eligible for inclusion. For each patient, a single stone specimen was selected for analysis, either obtained following spontaneous passage or retrieved during surgical or endourological procedures.

Stone specimens that were fragmented, incomplete, or unsuitable for accurate compositional analysis were excluded. Only patients with confirmed complete stone clearance and sufficient follow-up data were retained for outcome analysis.

Stone-free status was defined as the absence of residual fragments on post-procedural imaging or intraoperative assessment, according to the standard clinical practice of the treating team.

### 2.3. Data Collection and Clinical Assessment

Clinical and demographic data were obtained from institutional electronic medical records. Behavioral, dietary, medication, and comorbidity data were collected using a standardized clinical assessment questionnaire completed by all patients at the time of evaluation. The questionnaire included items on usual fluid intake, hydration pattern, selected dietary habits, current medication use, and relevant medical history. For analysis, these variables were grouped using predefined clinical categories. Low fluid intake was defined as reported daily fluid intake below 2 L/day, while irregular hydration was defined as inconsistent fluid intake throughout the day. Dietary variables, including salt intake, fruit and vegetable intake, sugary drink consumption, and red meat intake, were categorized according to the thresholds used in the clinical questionnaire.

Patients with a documented history of previous stone episodes or prior stone-related interventions were classified as having recurrent lithiasis at baseline.

**Figure 1:**
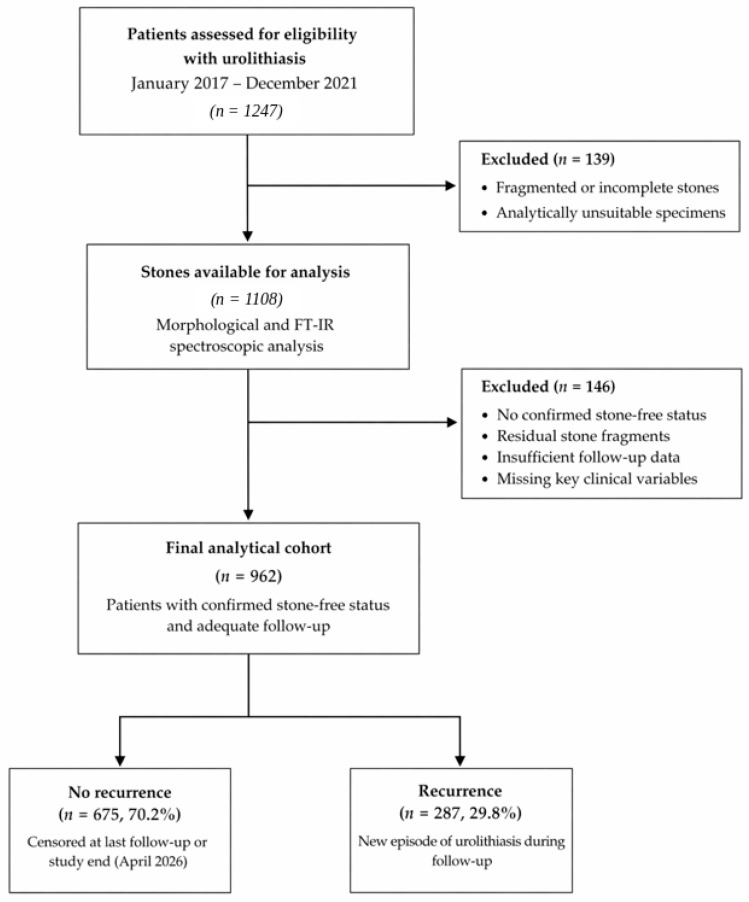
Flow diagram of patient selection and study cohort formation.

Follow-up information was obtained exclusively from the institutional electronic system, based on subsequent patient presentations within the same center. The patient selection process is summarized in Figure 1.

### 2.4. Stone Analysis

All retrieved calculi were subjected to systematic morphological and spectroscopic analysis. Morphological examination was performed using a stereomicroscope (Olympus, Hachioji, Tokyo), evaluating structural organization, color, and characteristic morphological features associated with lithogenic mechanisms.

Spectroscopic analysis was conducted using Fourier-transform infrared (FT-IR) spectroscopy (Bruker Alpha II, Bruker, Ettlingen, Germany), following standardized procedures. Spectral interpretation was performed according to the Daudon classification system.

Each stone was classified based on its predominant crystalline component, defined as the component with the highest relative proportion.

### 2.5. Outcome Definition and Follow-Up

The primary outcome was stone recurrence. Recurrence was defined as a new episode of urolithiasis occurring after confirmed stone-free status and documented within the institutional electronic medical record system. Recurrence events were identified only when patients re-presented to the same institution during follow-up. Exposures were measured before outcome.

Follow-up started at the date of confirmed stone clearance and continued until the earliest occurrence of recurrence, last recorded institutional contact, or the study closure date in April 2026. Patients without documented recurrence were treated as censored observations.

Given the tertiary nature of the center, follow-up relied on patient re-presentation within the same institutional framework, and events occurring outside the institution could not be fully captured.

### 2.6. Variables of Interest

The analysis included demographic characteristics, predominant stone composition, prior stone history, selected comorbidities, medication use, hydration-related variables, and selected dietary and behavioral factors. Clinical variables were defined from documented medical records, while behavioral and dietary variables were derived from the standardized clinical assessment questionnaire and categorized according to predefined clinically relevant thresholds.

Suboptimal adherence to preventive recommendations was defined as patient-reported non-adherence to previously recommended preventive measures, as documented in the standardized clinical questionnaire. This included inadequate adherence to advised fluid intake, dietary or lifestyle recommendations, or prescribed preventive medication, when applicable. This variable was treated as a pragmatic clinical indicator and not as a validated quantitative adherence score.

Variables were considered for analysis if they were clinically or epidemiologically relevant to urolithiasis recurrence, routinely available in the standardized assessment, and sufficiently complete for retrospective analysis.

### 2.7. Statistical Analysis

Statistical analysis was performed using SPSS software, version 27.0 (IBM Corp., Armonk, NY, USA). Categorical variables were summarized as frequencies and percentages and compared using the chi-square test or Fisher’s exact test, as appropriate. Continuous variables were reported as mean ± standard deviation or median with interquartile range, according to their distribution.

Recurrence-free survival was estimated using the Kaplan–Meier method, and differences between groups were assessed using the log-rank test. Independent predictors of recurrence were evaluated using Cox proportional hazards regression analysis. The proportional hazards assumption was assessed using Schoenfeld residuals and by visual inspection of survival curves.

Variables entered the multivariable Cox proportional hazards regression model were selected a priori based on clinical plausibility, epidemiological relevance to urolithiasis recurrence, routine availability in the medical records or standardized assessment questionnaire, and sufficient completeness for retrospective analysis. Candidate variables included demographic characteristics, predominant stone composition, previous stone history, selected comorbidities, hydration-related factors, and selected dietary and behavioral variables. These variables were considered because they represent established or clinically plausible contributors to stone recurrence risk, including baseline patient susceptibility, metabolic or systemic predisposition, prior recurrence tendency, and modifiable preventive factors.

To reduce model overfitting and avoid inclusion of variables with limited statistical contribution, candidate predictors were first evaluated in univariable Cox regression analyses. Variables associated with recurrence at *p* < 0.10 in univariable analysis were subsequently entered into the multivariable Cox regression model. This threshold was chosen to allow potentially relevant predictors to be retained for adjustment while maintaining a parsimonious model appropriate for the available sample size and number of recurrence events. Results were reported as hazard ratios (HRs) with 95% confidence intervals (CIs). A two-sided *p*-value < 0.05 was considered statistically significant.

### 2.8. Ethical Considerations

The study protocol was approved by the Institutional Research Ethics Committee of the “Dr. C. I. Parhon” Hospital—18/08.03.2022, approval date: 6 May 2022. All data were anonymized prior to analysis, and the study was conducted in accordance with applicable ethical standards.

## 3. Results

### 3.1. Study Population and Follow-Up

During the study period, 1247 adult patients with urolithiasis were evaluated. After exclusion of fragmented or analytically unsuitable specimens, 1108 stones were available for morphological and FT-IR spectroscopic analysis. For outcome analyses, patients without confirmed stone-free status, those with residual fragments, and those lacking sufficient follow-up data within the institutional system were excluded, resulting in a final analytical cohort of 962 patients.

The mean age of the cohort was 52.8 ± 13.9 years, and 568 patients (59.0%) were male. Median follow-up after confirmed stone clearance was 37 months (IQR 19–63 months).

During follow-up, 287 patients (29.8%) developed at least one institutionally documented recurrence, while 675 patients (70.2%) remained recurrence-free within the available records.

Patients with recurrence were older and had longer observed follow-up, whereas no significant differences were observed for sex distribution or place of residence, as shown in Table 1.

### 3.2. Stone Composition in the Overall Cohort

As detailed in Table 2, calcium oxalate stones were the predominant category (56.9%), with COM representing the largest subgroup, followed by COD. Uric acid and urate-mixed stones together accounted for approximately 27% of cases, whereas carbapatite and struvite stones were less frequent but clinically relevant.

### 3.3. Stone Composition According to Recurrence Status

As shown in Table 3, stone composition differed between patients with and without recurrence. COM stones were more frequent among patients without recurrence, whereas uric acid and urate-mixed stones were more prevalent among those with recurrence. Differences for COD and carbapatite were minimal, while struvite stones showed a non-significant trend toward higher recurrence.

### 3.4. Lifestyle, Clinical, and Behavioral Characteristics

Several clinical and behavioral characteristics differed between groups, although the observed effect sizes were generally modest, as detailed in Table 4. Low fluid intake and prior stone history emerged as the strongest correlates of recurrence, whereas most dietary variables showed weaker or borderline associations. Other factors did not differ significantly between groups.

### 3.5. Recurrence-Free Survival

**Figure 2:**
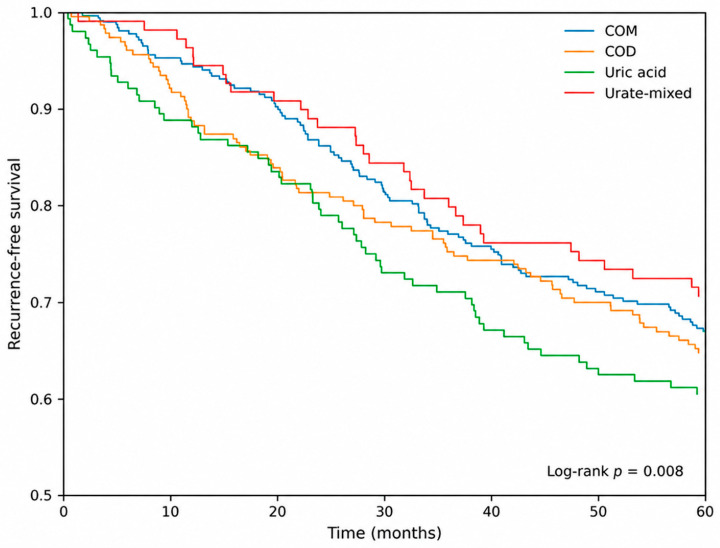
Kaplan–Meier curves for recurrence-free survival according to predominant stone composition.

Kaplan–Meier analysis demonstrated significant differences in recurrence-free survival according to predominant stone composition (log-rank *p* = 0.008), as illustrated in Figure 2. Patients with COM stones exhibited the most favorable recurrence-free survival, whereas those with uric acid stones showed a higher risk of earlier recurrence. Patients with urate-mixed stones demonstrated an intermediate pattern, while curves for COD and carbapatite overlapped substantially, indicating no clear difference between these groups.

Overall recurrence-free survival rates in the cohort were 90.2% at 1 year, 74.1% at 3 years, and 61.5% at 5 years.

Lower recurrence-free survival was also observed among patients with fluid intake below 2 L/day, with divergence between curves becoming more apparent after approximately 24 months of follow-up.

### 3.6. Univariable Cox Regression Analysis

In univariable Cox regression analysis, several variables showed associations with recurrence at the predefined inclusion threshold, as detailed in Table 5. Increasing age was associated with a higher risk of recurrence. In terms of stone composition, uric acid stones were linked to a significantly increased risk compared with COM, while urate-mixed stones showed a weaker association. No clear differences were observed for COD or carbapatite stones.

Among clinical and behavioral factors, low fluid intake was associated with recurrence, whereas irregular hydration, low fruit and vegetable intake, and a history of recurrent urinary tract infection showed only modest or borderline associations. Previous stone episodes and poor adherence to preventive recommendations were also associated with recurrence.

Other variables, including sex, place of residence, dietary factors such as salt and red meat intake, and most comorbidities, were not clearly associated with recurrence in univariable analysis. Variables with *p* < 0.10 were subsequently included in the multivariable Cox model.

### 3.7. Multivariable Cox Regression Analysis

Variables with *p* < 0.10 in univariable analyses were included in the multivariable model. After adjustment, age, uric acid stones, low fluid intake, and previous stone history remained independently associated with recurrence, while Suboptimal adherence to preventive recommendations showed a borderline association. Other variables did not retain statistical significance, as detailed in Table 6. The proportional hazards assumption was checked using Schoenfeld residuals. The global test was not significant, suggesting no clear violation of this assumption in the final Cox model (*p* = 0.554).

## 4. Discussion

In our present study, recurrence in urolithiasis was evaluated by integrating stone composition with clinical and behavioral variables within a time-to-event framework. Approximately one-third of patients experienced recurrence during follow-up. This was not uniformly distributed across groups, as patients with uric acid stones showed a higher risk of recurrence, whereas those with COM stones tended to have more favorable outcomes.

The association between stone composition and recurrence has been reported in previous studies. Vaughan et al. identified uric acid and infection-related stones among the factors associated with an increased likelihood of subsequent symptomatic episodes [9,10]. In our cohort, uric acid stones were more frequent among recurrent cases and remained associated with recurrence after adjustment. However, the magnitude of this effect was moderate, suggesting that composition alone does not fully explain recurrence patterns.

A notable finding in our study is the variability in recurrence across patients. Even within similar compositional groups, outcomes differed considerably. This observation is consistent with previous reports showing that recurrence risk increases progressively with the number of prior stone episodes, reflecting a cumulative lithogenic burden [7,11,12]. In our analysis, a previous stone episode remained an independent predictor of recurrence, supporting this interpretation.

Related evidence comes from registry-based studies. Li et al. reported that patients with early-onset urolithiasis are more likely to present with multiple recurrences and a more aggressive disease course [5,13]. Although age at first episode was not specifically addressed in our analysis, the association observed between prior stone history and recurrence is consistent with the concept of a persistent individual predisposition.

From an epidemiological perspective, recurrence in urolithiasis should be viewed not as a single outcome but as a dynamic process that evolves over time. Population-based studies have shown that recurrence risk is not constant but increases with cumulative exposure to lithogenic factors and prior disease burden [10]. In this context, time-to-event analysis provides a more appropriate framework than simple proportion-based measures, as it captures both the occurrence and timing of recurrence.

The recurrence rates observed in the present cohort are consistent with previously reported data. It is generally estimated that 30–50% of patients will experience recurrence within 10 years, although this varies considerably between populations [14]. In our study, recurrence occurred in approximately one-third of patients over a median follow-up exceeding three years, which is compatible with these estimates when accounting for differences in observation time.

Importantly, epidemiological studies have also highlighted the wide dispersion of individual risk. Forbes et al. showed that predicted recurrence risk can vary substantially between patients, ranging from low to very high probabilities depending on clinical characteristics [14,15]. This variability is also reflected in our findings, where no single variable fully accounted for recurrence risk, and effect sizes remained moderate even for statistically significant predictors. While uric acid stone composition, previous stone episode, and low fluid intake were independently associated with recurrence, none of these factors alone is sufficient to define individual recurrence risk. Their practical value is therefore mainly in supporting cumulative risk assessment, where compositional, clinical, and behavioral factors are considered together to guide follow-up intensity and preventive counseling.

The role of modifiable factors should be interpreted within this broader epidemiological framework. Low fluid intake was independently associated with recurrence, reinforcing the importance of urinary dilution in reducing lithogenic risk [16,17]. At the population level, modifiable exposures such as fluid intake may yield greater preventive impact than compositional factors [18,19]. Morpho-spectroscopic studies have also emphasized the importance of the metabolic context in which stones form. Features such as urine concentration, crystallization environment, and the presence of specific crystalline phases may provide indirect information on lithogenic conditions and recurrence propensity. Therefore, stone composition should be interpreted together with clinical and behavioral factors, rather than as an isolated marker of recurrence risk [2,20].

By contrast, dietary variables such as salt intake and fruit and vegetable consumption did not retain statistical significance after adjustment. This may reflect both methodological and biological factors. From an epidemiological standpoint, dietary exposures are difficult to measure accurately and are often subject to misclassification. In addition, their effects are likely mediated through complex metabolic pathways rather than acting as independent risk factors [19,20]. Several limitations should be considered. First, the retrospective design limits causal interpretation and carries an inherent risk of selection bias. The final cohort included only patients with confirmed stone-free status and sufficient follow-up data. This was necessary to separate recurrence from residual disease, but it may have favored patients with more complete institutional records or better healthcare engagement. Since comparable data were not systematically available for excluded patients, their characteristics could not be assessed in detail.

Second, recurrence was captured only when patients re-presented within the same institutional system. Events diagnosed or treated elsewhere, as well as asymptomatic or mildly symptomatic episodes not leading to re-presentation, may therefore have been missed. This could have led to an underestimation of recurrence and to follow-up bias. The longer follow-up observed in patients with institutionally documented recurrence may also have increased the chance of detecting events in this group. Although Kaplan–Meier and Cox regression methods account for unequal follow-up and censoring, they cannot fully account for differences in follow-up intensity or patterns of re-presentation. For this reason, the recurrence estimates should be interpreted as institutionally documented rates rather than population-level rates.

Third, follow-up imaging was performed as part of routine care, without a single standardized imaging protocol. Differences between imaging methods may have influenced recurrence detection, especially for small or asymptomatic stones. Behavioral and dietary data were collected using the same clinical questionnaire for all patients, but these variables were self-reported and may be affected by reporting bias or exposure misclassification. Data on other potentially relevant lifestyle or socioeconomic factors, including smoking status, alcohol consumption, BMI, and educational attainment, were not available in a sufficiently complete and standardized manner, limiting adjustment for these potential confounders. Finally, detailed metabolic evaluation was not uniformly available. In particular, standardized data on idiopathic hypercalciuria were not available for the whole cohort, which prevented a reliable comparison across stone-composition subgroups and limited adjustment for underlying biochemical risk factors.

## 5. Conclusions

In this cohort, recurrence of urolithiasis was associated with both stone composition and clinical or behavioral factors. Uric acid stones, previous stone history, and low fluid intake were linked to a higher risk of recurrence, although the observed effect sizes were moderate.

These findings support a cumulative approach to recurrence risk assessment. In clinical practice, patients with uric acid stones, a history of previous stone episodes, or low fluid intake may require closer follow-up and more targeted preventive counseling, particularly regarding hydration. However, recurrence risk cannot be defined by a single factor, and preventive strategies should be adapted to the broader clinical context of each patient.

Future prospective studies using standardized follow-up protocols, consistent imaging strategies, and detailed metabolic evaluation are needed to better quantify recurrence risk and to validate individualized risk stratification models in patients with urolithiasis.

## Figures and Tables

**Table 1:** Baseline characteristics of the final analytical cohort.

Variable	No Recurrence (*n* = 675)	Recurrence (*n* = 287)	Total (*n* = 962)	*p*-Value
Age, years (mean ± SD)	51.6 ± 14.2	55.5 ± 12.8	52.8 ± 13.9	0.002
Male sex, n (%)	394 (58.4)	174 (60.6)	568 (59.0)	0.53
Female sex, n (%)	281 (41.6)	113 (39.4)	394 (41.0)	—
Urban residence, n (%)	410 (60.7)	180 (62.7)	590 (61.3)	0.57
Follow-up, months (median [IQR])	33 [17–57]	45 [26–70]	37 [19–63]	<0.001

**Table 2:** Overall biochemical stone composition in the final cohort.

Stone Type	*n* (%)
COM (calcium oxalate monohydrate)	318 (33.1)
COD (calcium oxalate dihydrate)	230 (23.9)
Uric acid	152 (15.8)
Urate-mixed	109 (11.3)
Carbapatite	103 (10.7)
Struvite	44 (4.6)
Cystine and Brushite Stones	6 (0.6)

**Table 3:** Stone composition according to recurrence status.

Stone Type	No Recurrence (*n* = 675)	Recurrence (*n* = 287)	*p*-Value
COM	248 (36.7)	70 (24.4)	<0.001
COD	165 (24.4)	65 (22.6)	0.56
Uric acid	92 (13.6)	60 (20.9)	0.006
Urate-mixed	69 (10.2)	40 (13.9)	0.09
Carbapatite	73 (10.8)	30 (10.5)	0.91
Struvite	24 (3.6)	20 (7.0)	0.07
Cystine and Brushite Stones	4 (0.6)	2 (0.7)	0.88

The “Others” category includes cystine and brushite stones.

**Table 4:** Lifestyle, clinical, and behavioral characteristics according to recurrence status.

Variable	No Recurrence (*n* = 675)	Recurrence (*n* = 287)	*p*-Value
Low fluid intake (<2 L/day)	272 (40.3%)	148 (51.6%)	0.002
Irregular hydration	235 (34.8%)	118 (41.1%)	0.07
High salt intake	202 (29.9%)	96 (33.4%)	0.29
Low fruit/vegetable intake	252 (37.3%)	118 (41.1%)	0.26
Sugary drinks ≥ 3/week	150 (22.2%)	75 (26.1%)	0.21
Red meat ≥ 4/week	168 (24.9%)	79 (27.5%)	0.41
Antihypertensive treatment	109 (16.1%)	62 (21.6%)	0.05
Antidiabetic treatment	61 (9.0%)	33 (11.5%)	0.26
Recurrent UTI history	66 (9.8%)	39 (13.6%)	0.08
Previous stone episode	178 (26.4%)	114 (39.7%)	<0.001
Suboptimal adherence to preventive recommendations	161 (23.9%)	90 (31.4%)	0.02

**Table 5:** Univariable Cox regression analysis of predictors of recurrence.

Variable	HR	95% CI	*p*-Value
Age (per 10 years)	1.19	1.08–1.31	0.001
Male sex	1.08	0.86–1.35	0.53
Urban residence	1.06	0.84–1.33	0.57
Stone composition			
COM	Reference	—	—
COD vs. COM	1.10	0.81–1.49	0.54
Uric acid vs. COM	1.73	1.29–2.31	<0.001
Urate-mixed vs. COM	1.42	1.01–1.98	0.04
Carbapatite vs. COM	1.08	0.73–1.60	0.69
Struvite vs. COM	1.31	0.79–2.18	0.29
Lifestyle and clinical factors			
Low fluid intake (<2 L/day)	1.47	1.19–1.82	<0.001
Irregular hydration	1.24	1.00–1.53	0.05
High salt intake	1.18	0.94–1.48	0.15
Low fruit/vegetable intake	1.26	1.02–1.55	0.03
Sugary drinks ≥ 3/week	1.11	0.87–1.41	0.40
Red meat ≥ 4/week	1.09	0.85–1.39	0.50
Antihypertensive treatment	1.23	0.96–1.58	0.10
Antidiabetic treatment	1.15	0.80–1.63	0.45
Recurrent UTI history	1.29	0.98–1.69	0.07
Previous stone episode	1.55	1.25–1.92	<0.001
Previous stone intervention	1.17	0.89–1.54	0.26
Poor adherence to preventive recommendations	1.34	1.07–1.67	0.01

**Table 6:** Multivariable Cox regression model for predictors of recurrence.

Variable	Adjusted HR	95% CI	*p*-Value
Age (per 10 years)	1.14	1.02–1.27	0.02
Uric acid vs. COM	1.58	1.16–2.15	0.004
Urate-mixed vs. COM	1.29	0.93–1.79	0.12
Low fluid intake (<2 L/day)	1.34	1.08–1.66	0.007
Irregular hydration	1.17	0.95–1.45	0.14
Recurrent UTI history	1.18	0.89–1.56	0.24
Previous stone episode	1.39	1.12–1.72	0.003
Suboptimal adherence to preventive recommendation	1.26	1.00–1.58	0.048

## Data Availability

The data that support the findings of this study are available from the corresponding author upon reasonable request.

## Abbreviations

The following abbreviations are used in this manuscript:

Abbreviation	Definition
COM	Calcium oxalate monohydrate
COD	Calcium oxalate dihydrate
UTI	Urinary tract infection
FT-IR	Fourier-transform infrared spectroscopy
HR	Hazard ratio
CI	Confidence interval
IQR	Interquartile range
KM	Kaplan–Meier
SD	Standad deviation
